# School Parks as a Community Health Resource: Use of Joint-Use Parks by Children before and during COVID-19 Pandemic

**DOI:** 10.3390/ijerph18179237

**Published:** 2021-09-01

**Authors:** Kevin Lanza, Casey P. Durand, Melody Alcazar, Sierra Ehlers, Kai Zhang, Harold W. Kohl

**Affiliations:** 1 Michael and Susan Dell Center for Healthy Living, School of Public Health in Austin, The University of Texas Health Science Center at Houston, Austin, TX 77030, USA; Harold.W.Kohl@uth.tmc.edu; 2 Michael and Susan Dell Center for Healthy Living, Department of Health Promotion & Behavioral Sciences, School of Public Health in Houston, The University of Texas Health Science Center at Houston, Houston, TX 77030, USA; Casey.P.Durand@uth.tmc.edu; 3 Parks and Recreation Department, Austin, Austin, TX 78704, USA; melody.alcazar@austintexas.gov (M.A.); sierra.ehlers@austintexas.gov (S.E.); 4 Department of Environmental Health Sciences, School of Public Health, University at Albany, State University of New York, Rensselaer, NY 12144, USA; kzhang9@albany.edu; 5 Department of Epidemiology, Human Genetics, and Environmental Sciences, School of Public Health in Austin, The University of Texas Health Science Center at Houston, Austin, TX 78701, USA; 6 Department of Kinesiology and Health Education, The University of Texas at Austin, Austin, TX 78712, USA

**Keywords:** Youth Physical Activity, shared-use parks, urban greenspace, social distancing, face masks, COVID-19 pandemic, Latinx, communities of color, low-income communities

## Abstract

Parks are settings for physical activity that can support the physical and mental health of children during the COVID-19 pandemic. We determined the impact of the pandemic on the use of joint-use parks outside of school hours by children in Austin, TX, United States. In autumn of 2019 and autumn of 2020 (i.e., before and during the COVID-19 pandemic), we used an adapted version of the System for Observing Play and Recreation in Communities to observe whether children aged 1–12 participated in physical activity (i.e., sedentary, light and moderate, vigorous) at three parks located at schools serving mostly economically disadvantaged Latinx families. In 2020, we also observed whether children maintained social distance and wore face coverings. Results of negative binomial regression modeling revealed the pandemic was associated with a 46% [95% CI: 20–63%] and 62% [95% CI: 39–76%] decrease in the number of girls and boys at parks, respectively, and a 42% [95% CI: 16–59%] and 60% [95% CI: 36–75%] decrease in the number of girls and boys engaging in physical activity, respectively (*p* < 0.01). In total, 60.6% of girls and 73.6% of boys were not social distancing, and 91.8% of the time no children wore masks. Interventions should be considered to safely reintroduce children to parks for health benefits during pandemics.

## 1. Introduction

Children may seem to be less impacted by the health challenges presented by Coronavirus Disease 2019 (COVID-19): researchers have estimated the susceptibility of children to COVID-19 is 43% of the susceptibility of adults [1], and surveillance in the United States has revealed children aged 0–17 constitute 19.3% of the US population yet only 11.9% of total COVID-19 cases and less than 0.1% of related deaths [2]. However, children have experienced a high burden of indirect health effects from the pandemic. Service closures, social isolation, parental stress, and the pandemic itself have been found to be associated with higher levels of anxiety, depression, obsessive-compulsive disorder symptoms, and behavioral problems in children [3,4,5]. Moreover, the pandemic has been found to disproportionately affect the mental health of children with low socioeconomic status, migration background, and limited living space [6].

Regarding movement behaviors of children, the pandemic has been found to associate with increased sedentary behavior and screen time, increased sleeping duration and decreased sleep quality, and decreased physical activity levels [7,8,9,10,11,12,13,14]. Regarding physical activity, children and adolescents aged 6–17 in Shanghai, China, reported an average reduction in time spent engaging in physical activity of 435 min per week during the pandemic (March 2020) compared to before the pandemic (January 2020) [14]. Two studies conducted in Spain have shown a reduction of 91 min per day and 102.5 min per week, respectively, of time children spent participating in physical activity during the enacted national confinement [10,11]. In a US-based study, researchers found 36% of parents and legal guardians reported their child had engaged in much less physical activity during the pandemic compared to February 2020 [9].

To combat the health impacts of the COVID-19 pandemic on children, access to greenspace has been recommended [15,16]. Greenspace has been identified as a relatively safe space during the pandemic: the odds of outdoor transmission of COVID-19 have been found to be 18.7 times lower compared with indoor transmission [17]. Exposure to greenspace has been found to associate with mental well-being, overall health, and cognitive development of children [18], and parks have been identified as places for physical activity, especially for youth [19]. Yet, greenspace is inequitably distributed, with communities of color and low-income communities disproportionately living in areas with less trees and vegetation [20]. Limited access to environments conducive to physical activity has been shown to partly contribute to residents in these communities engaging in less physical activity than others [21]. In 2015–2016, only 5.6% of Latinx students in 4th grade across Texas reported meeting the Physical Activity Guidelines for Americans of 60 min of physical activity daily, the lowest percentage of all racial and ethnic groups [22]. One strategy to increase access to greenspace and promote physical activity is to designate a school park as joint use, wherein a written agreement between a school and city or community entity permits shared use of the park (i.e., use limited to the school population during school hours and use open to the public outside of school hours).

Only a pair of studies have focused on the impact of the COVID-19 pandemic on the use of parks by children. While playgrounds were closed in Calgary, Canada, children’s play at parks and public spaces decreased by 52.7% and 53.7%, respectively [23]. In a study of US children aged 5–13, researchers estimated a decreased likelihood (OR = 0.47) of children performing physical activity at a park or trail during the pandemic (9). In all studies focused on the influence of the pandemic on children’s physical activity [7,8,9,10,11,12,13,14,23], researchers retroactively measured physical activity from before the pandemic by survey—instruments that are prone to bias [24].

To our knowledge, no studies have focused on adherence to COVID-19 protective health behaviors (e.g., practicing social distancing and wearing face coverings) of children while at parks. Several studies have assessed the level of adherence to COVID-19 protective health behaviors of adults [25,26,27,28,29], yet limited evidence exists for children. No studies have focused on whether children practiced social distancing during the pandemic. Regarding adolescents and young adults, one study has found 83.5% of adolescents aged 13–18 years in the US reported engaging in social distancing “either a lot or a great deal” [30], and another study has found 95% of American youth aged 14–24 reported social distancing to some extent [31]. The single US-based study that assessed whether children wore face coverings revealed 37% of individuals aged 2–30 wore face coverings in retail locations in Wisconsin [32]. The only other studies on whether children wore face coverings occurred during the first months of the pandemic in China where face masks were mandatory in public spaces [33,34]. In those studies, 88.6% of children (median age: 12.0 years) reported wearing masks always [35], and 51.6% of children aged 6–13 reported good mask-wearing behavior [36].

In this study, we used direct observation to determine the relations between the COVID-19 pandemic and the presence and physical activity levels of children outside of school hours at joint-use parks in low-income communities of color. As a secondary aim, we assessed whether children observed at these parks practiced the COVID-19 protective health behaviors of maintaining social distance and wearing face coverings. In testing these aims, we found that less children visited parks and were physically active at parks during the pandemic compared to before the pandemic. We also found that most children were not maintaining social distance or wearing face masks during the pandemic.

## 2. Materials and Methods

### 2.1. Study Setting

This serial cross-sectional study took place at three joint-use elementary school parks in the City of Austin, Texas, United States, during non-school hours for two September weeks and one November week—all weekdays and one randomly selected weekend day per week—in both 2019 and 2020 (n = 36 days). Based on the Youth Physical Activity Timing, How, and Setting (Y-PATHS) framework [37], this study focused on physical activity of children during school days outside of school hours and non-school days (i.e., Timing), categorized as free play and organized (i.e., How), and occurring at schools (i.e., Setting). The study was part of the Green Schoolyards Project (Robert Wood Johnson Foundation, grant number 76576), which aimed to determine how green features (i.e., trees, gardens, and nature trails) in school parks impacted heat index within parks, and physical activity levels and socioemotional well-being of predominantly Latinx children from economically disadvantaged families. Further detail on the design and methods of the Green Schoolyards Project have been published [38]. Prior to undertaking project activities, we received approval of project protocols by the school district and institutional review board at The University of Texas Health Science Center at Houston (HSC-SPH-19-0502).

**Figure 1:**
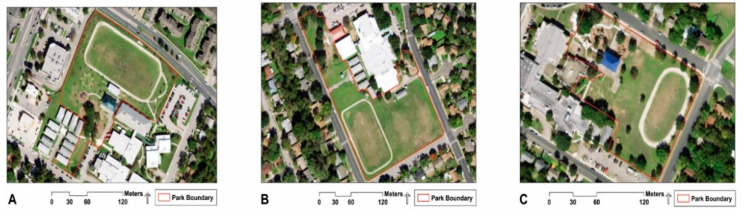
(**A**) Intervention park, (**B**) low-green park, and (**C**) high-green park.

The three study parks had different levels of green features (Figure 1): the “intervention park” had added green features (i.e., saplings, wildflower meadow, and nature trail); the “low-green park” had relatively low amounts of historical green features (i.e., trees); the “high-green park” had relatively high amounts of historical green features (i.e., trees, wildlife habitat garden, and nature trail). Using i-Tree Canopy [39], we calculated the amount of tree canopy at the intervention, low-green, and high-green parks to be 8.5% (standard error = 1.97), 11.5% (standard error = 2.26), and 22.5% (standard error = 2.95), respectively.

The study parks had several similarities. First, each park had a joint-use agreement between the Austin Parks and Recreation Department and Austin Independent School District permitting public use of school grounds outside the time between 7:00 and 16:00. Second, each park was located at an elementary school serving populations greater than 85% economically disadvantaged Latinx. Third, each park was of similar size (i.e., intervention = 21,448 m^2^; low-green = 27,923 m^2^; high-green = 16,187 m^2^) and had equivalent facilities (i.e., playgrounds, soccer fields, multipurpose fields, running tracks, and basketball courts with artificial shade structures). Lastly, each park was located in a ZIP code with Nature Factor Scores—defined as the sum of Nature Factor Ratings (i.e., park acreage rating, Trust for the Public Land land use rating, National Recreation and Parks Association park status rating, and tree canopy rating) of all parks within a ZIP code, with higher scores corresponding to higher levels of nature—that were relatively low (i.e., intervention = 121; low-green = 118; high-green = 198) compared to those of other ZIP codes (n = 53; minimum = 0; maximum = 712; mean = 150; standard deviation = 143) [40,41].

### 2.2. COVID-19 Pandemic in Texas

**Figure 2:**
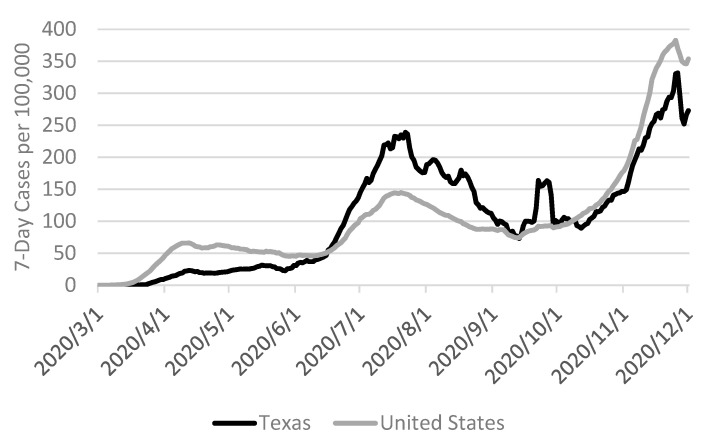
Daily trends in seven-day cumulative incidence rate of COVID-19 cases in Texas and the United States per 100,000 population [43].

On 13 March 2020, the President of the United States declared the COVID-19 pandemic a national emergency for all states, tribes, territories, and the District of Columbia [42]. Between then and the final study days in November 2020, daily trends in seven-day cumulative incidence rate of COVID-19 cases reported to the US Centers for Disease Control and Prevention were comparable between the State of Texas and the United States with some notable distinctions (Figure 2). From 16 June–9 October 2020, cases in the last seven days per 100,000 individuals were higher in Texas than the US, with the largest percentage difference (50.4%; 238.8 versus 142.7 seven-day cases per 100,000) occurring on 22 July 2020 [43].

During the 18 study days across September and November 2020, the COVID-19 orders in effect remained the same. On 14 August 2020, the Mayor of Austin extended the existing Stay Home, Mask, and Otherwise Be Safe order in Austin to 16 August–15 December 2020. Specific to our focus on children and use of public parks, the order established all persons must practice social distancing—at least a six-foot distance from other individuals—except when in the presence of members in their own household, when passing someone is incidental and momentary, and when dining in groups of 10 or less. Second, the order established all persons must wear a face covering when outside their residence unless the person is younger than 10 years of age, or the person is engaging in physical activity outdoors and maintaining social distance from those in other households. The order stated that parents and guardians of children younger than 10 were responsible for ensuring these children adhered to social distancing and face covering guidelines. Lastly, the order prohibited outdoor gatherings of more than 10 persons [44]. 

**Figure A1:**
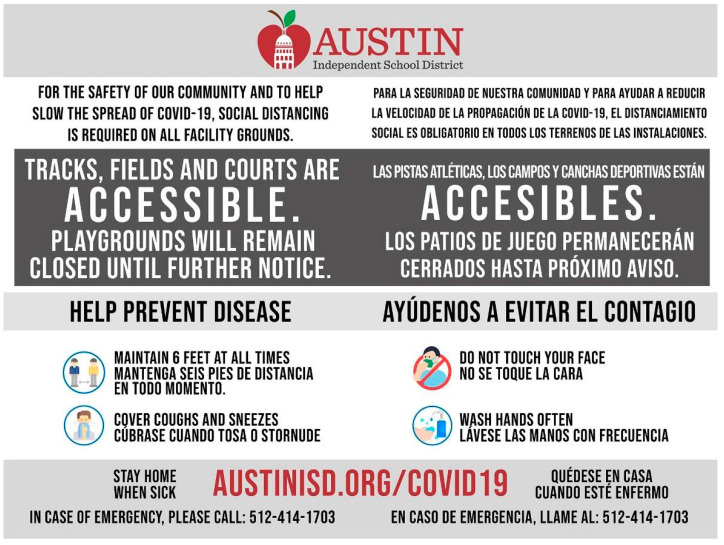
Signage of COVID-19 requirements installed at joint-use parks in Austin, TX, USA.

In addition to the city-wide Stay Home, Mask, and Otherwise Be Safe order, Austin Independent School District developed COVID-19 regulations for its facilities that went into effect on 27 April 2020 and remained in place throughout the study period. Signs installed at joint-use school parks stated that social distancing was required, tracks, fields, and courts were accessible but playgrounds would remain closed until further notice, and persons can prevent disease by practicing several protective health behaviors (i.e., maintain six feet at all times, cover coughs and sneezes, no face touching, wash hands often, and stay home when sick) (Figure A1) [45]. Signage did not include content about face coverings. Playgrounds, albeit officially closed, were still accessible and not policed for compliance. 

### 2.3. Measurement of Park Use by Children

**Figure A2:**
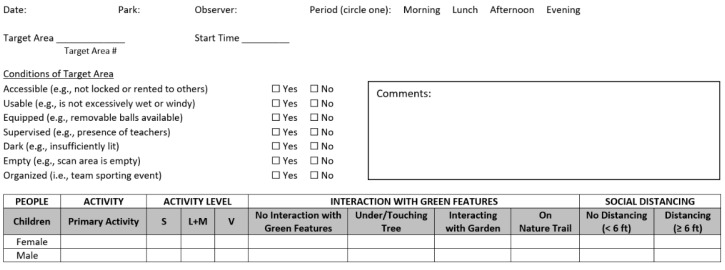
System for Observing Play and Recreation in Communities (SOPARC) data collection form.

To measure the daily number of children present at each study park when parks were publicly accessible and the number of these children engaging in physical activity, we used a validated tool for direct observation called the System for Observing Play and Recreation in Communities (SOPARC) [46]. Per SOPARC protocol, we first divided each park into target areas (i.e., intervention = 21; low-green = 15; high-green = 21), which are sites intended for physical activity such as playgrounds, basketball courts, and soccer fields. Then at four times per study day (i.e., 7:00, 12:00, 16:00, and 18:00), trained staff—in pairs for interrater reliability—walked from target area to target area to observe the conditions and the number of female and male users and their physical activity levels (i.e., sedentary, light-to-moderate intensity, or vigorous intensity) at each target area and record observations on a data collection form (Figure A2). We adapted SOPARC to only observe children aged 1–12 at each target area and their physical activity levels. We defined children engaging in physical activity as those who were recorded as “light-to-moderate” or “vigorous.” In addition, we only focused on the two data collection times in our study when parks were open to community members (i.e., 16:00 and 18:00) since children’s use of parks during school hours was dependent on school schedules for recess and other programmed activities. 

### 2.4. Measurement of Variables That May Impact Park Use by Children

Children’s park use may be influenced by a number of factors, including the exposure variable of interest in this study: whether or not the study day occurred during the COVID-19 pandemic (1 = during pandemic, 0 = not during pandemic). We developed another temporal variable for weekends (weekend day = 1; weekday = 0) because children’s park use has been found to differ between weekend days and weekdays [47]. Since adverse weather conditions—precipitation, extreme heat, and high levels of relative humidity—have been shown to exhibit negative correlations with individuals’ park use and physical activity levels [48,49,50], we developed two weather variables: rain (1 = rain; 0 = no rain) during SOPARC observations and daily average heat index (°C) from 16:00 to 18:30. Study staff recorded in the Comments section of the SOPARC data collection form whether it was raining while conducting observations (Figure A2). We measured heat index by installing 10 HOBO MX2302A external air temperature/relative humidity sensor data loggers (Onset Computer Corporation, Bourne, MA, USA) at each park, per previous studies [51,52]. We then calculated heat index using equations from the US National Weather Service [53].

### 2.5. Measurement of COVID-19 Protective Health Behaviors by Children

We adapted the SOPARC tool to measure whether children aged 1–12 were practicing social distancing and wearing face coverings (Figure A2). To calculate the daily number of children who practiced social distancing, we added two separate observational scans to determine the number of female and male children who were within six feet of another person and who were six feet or more from another person, per city and school district orders [44,45]. When conducting fieldwork, trained staff brought a six-foot-long PVC pipe to estimate the requisite social distance. For the measurement of children wearing face coverings, study staff input—in the Comments section of the data collection form—the number of children (i.e., all, some, or none) that properly wore face coverings over their mouth and nose. We only measured whether children were practicing social distancing and wearing face coverings when more than one child was present in a target area. 

As with measuring park use of children, we only focused on the two data collection times when parks were open to community members (i.e., 16:00 and 18:00) because the school district had required all students to wear face coverings while on school grounds during school hours [54,55]. During study days in 2020, only one of the trained staff members—each of whom collected data in 2019—collected data per park to reduce the risk of COVID-19 transmission and because COVID-19 policies by the school district limited the number of persons on campus.

### 2.6. Statistical Analyses

We first calculated Cohen’s kappa statistic to assess interrater reliability of SOPARC observations for children’s physical activity levels conducted by pairs of study staff. Next, we calculated summary statistics (i.e., mean, standard deviation, percentage of total, and count), which we aggregated across the three study parks and stratified by before and during the COVID-19 pandemic. We then used negative binomial regression modeling to estimate the relations between the COVID-19 pandemic and the daily number of children observed at parks outside of school hours and the number of these children engaging in physical activity, stratified by sex. We selected negative binomial regression for its application for modeling outcome variables consisting of over-dispersed count data [56]. Final models included adjustments for individual parks, weekends, rain during observations, and average heat index from 16:00 to 18:30. Lastly, we calculated the percentage of female and male children who were maintaining social distance (at least six feet from another) and wearing face coverings when more than one child was present in a park’s target area. Statistical analyses were completed in Stata 15.1 (StataCorp, College Station, TX, USA).

## 3. Results

In our assessment of interrater reliability of SOPARC observations conducted over the 18 study days in 2019, Kappa values ranged from 0.91 to 1.00, signifying “Almost Perfect” agreement between raters [57]. Since the same study staff from 2019 were retrained and collected data for the 18 study days in 2020, we expected SOPARC observations in 2020—collected by one staff member—to be a similar level of reliability. 

At the three joint-use parks outside of school hours for the 18 study days during the COVID-19 pandemic, the average number of female and male children observed was 2.74 and 6.29 lower, respectively, compared to before the pandemic (Table 1). Both before and during the pandemic, we observed most children participated in light-to-moderate intensity physical activity (before: 54.7% of females and 55.6% of males; after: 47.9% of females and 43.4% of males) compared to other intensity levels, and lower proportions of females participating in physical activity than males (before: 68.5% of females and 75.5% of males; after: 72.4% of females and 74.7% of males). A larger proportion of both sexes participated in vigorous intensity physical activity during the pandemic (female = 24.5%; male = 31.3%) than before the pandemic (female = 13.8%; male = 19.9%). Only four observations were impacted by rain, and the daily average heat index was 3.74 °C higher while collecting data before the pandemic compared to during the pandemic.

In fully adjusted regression models (Table 2), we found the COVID-19 pandemic was associated with a 46% [95% CI: 20–63%] and 62% [95% CI: 39–76%] decrease in the number of female and male children at parks outside of school hours, respectively (*p* < 0.01). In addition, the pandemic was associated with a 42% [95% CI: 16–59%] decrease in the number of female children engaging in physical activity, compared to a 60% [95% CI: 36–75%] decrease in male children (*p* < 0.01). When more than one child was present in a park’s target area, 60.6% of female children and 73.6% of male children observed were not maintaining social distance of at least six feet from another, and 91.8% of the time no children wore masks (Table 3).

## 4. Discussion

In this study, we determined that the COVID-19 pandemic was associated with significant declines in the number of overall children and number of physically active children at joint-use parks outside of school hours. These findings corroborate those in the body of literature that revealed the pandemic was associated with decreased physical activity levels of children [7,8,10,11,12,13,14], and in the few studies conducted that found the pandemic was associated with a reduction in trips to parks and children participating in physical activity at parks [9,23,58].

We posit the decline in the number of children and number of physically active children at joint-use parks during the pandemic was due to the COVID-19 order, changes in physical activity behavior of children, and impacts of the pandemic on guardians of children. Fewer children may have visited joint-use parks because of park signage stating playgrounds were closed and the loss of structured activities such as team sport practices. During the pandemic, children may have participated in less overall physical activity and/or shifted their physical activity elsewhere. Researchers have found children to be more likely to perform physical activity at home or in the garage (OR = 2.49; [95% CI: 1.35–4.60]) or on sidewalks and roads in their neighborhood (OR = 1.92; [95% CI: 1.04–4.60]) during the pandemic [9]. Additionally, guardians may not bring children to parks or allow them to travel there due to fear of COVID-19 transmission. Children of parents who expressed anxiety about COVID-19 have been shown to travel to parks less and be more likely to spend two or more hours per day on the computer or playing video games compared with children of less anxious parents [23]. Lastly, parents have discussed the lack of time and resources to bring children to parks during the pandemic, and the reduction in their children’s independent mobility because of concern over compliance with social distancing guidelines [59]. The COVID-19 pandemic may have magnified the resource constraints of residents of low-income communities and resulted in the opposite use of greenspace than residents of other communities: a park and trail system located in wealthier communities in Austin experienced increases in usage of 300% and 20%, respectively, during the pandemic compared to before the pandemic [60,61]. 

Although we found an overall decline in the number of children participating in physical activity at joint-use parks outside of school hours during the pandemic, we did find a 10.7% and 11.4% increase in the proportion of female and male children, respectively, participating in vigorous intensity physical activity compared with before the pandemic. This higher proportion may be attributed to children spending more time being sedentary and less time visiting parks during the pandemic [7,9,10,11,12,13], potentially inducing more intense play when visiting parks. Additionally, parents whose children typically engage in vigorous intensity physical activity may be more motivated to accompany their children to parks during the pandemic to accommodate their children’s penchant for play.

Regarding COVID-19 protective health behaviors, we observed that most children at joint-use parks outside of school hours did not maintain social distance or wear a face covering. Low levels of adherence to social distancing of observed children were different from the high levels of adherence to social distancing of adolescents and young adults in the US [30,31]. Our finding that most children did not wear face coverings was comparable to that of a US-based study of individuals aged 2–30 [32].

Among the potential reasons for limited adherence to COVID-19 protective health behaviors were that children and their guardians might have believed COVID-19 was not a health concern warranting behavior change and/or risk of viral transmission was lower outdoors. In addition, evolving and competing COVID-19 orders at both the city- and school district-level may have caused confusion: in a study of 944 youth aged 14–24 across the United States, 19% of youth reported misconceptions about social distancing guidelines as a reason for non-compliance [31]. Limited compliance with social distancing guidelines may have also been related to the type and location of park amenities. We observed children to be attracted to playgrounds and basketball courts, two amenities that permit multiple users simultaneously and are located near one another at all three parks; children using these amenities may have a higher likelihood of close contact with other park users. Adherence to social distancing may have also been impacted by games (e.g., tag), sports (e.g., soccer), and activities (e.g., socializing) that require more than one participant. 

Our finding that most children did not wear masks at parks may have been related to children being near only members of their household, masks being uncomfortable while engaging in physical activity, and/or adherence to global recommendations. Children aged 8–11 have been shown to remove masks because of the perceived difficulty in breathing and discomfort from overheating, especially when running compared to walking [62]. In August 2020, the World Health Organization and United Nations Children’s Fund had advised that children aged 5 and under should not be required to wear masks, and the decision for children aged 6–11 to wear masks is based on six factors: (1) whether there is widespread transmission in the area; (2) ability of child to wear the mask safely and correctly; (3) access to masks; (4) adult supervision; (5) impact of wearing a mask on learning and development; (6) settings where others are at high risk of developing serious illness [63]. 

Study results are particularly concerning because observations occurred at parks located at schools serving mostly economically disadvantaged Latinx students, a group shown to be less physically active compared to white students and facing multiple health inequities that existed prior to the pandemic due to historical discriminatory policies (e.g., redlining) and ongoing disinvestment [22,64,65,66]. A decline in park use by Latinx children during the pandemic may widen disparities in physical activity if Latinx children are not compensating in other settings. Further, the low adherence to COVID-19 protective behaviors observed in this study may serve as evidence for the higher COVID-19 contraction rate found among low-income and Latinx populations in the US [67].

Based on the observed decline in children’s use of joint-use parks outside of school hours during the pandemic and low adherence to COVID-19 protective behaviors while using parks, we recommend that city officials and school districts work together to promote the safe use of joint-use parks during the pandemic. Clear and consistent messaging on COVID-19 orders through multiple avenues (e.g., physical signage, government website, and information outlets used by schools) could support comprehension of park accessibility and rules. Youth sports organizations that utilize joint-use parks can adjust sport activities to reduce physical interactions and sharing of equipment while considering what is practical, acceptable, and tailored to the needs of the community [68]. Lastly, joint-use parks can have dedicated park times based on age group or entry allocation systems that—combined with smartphone apps or drones—monitor and manage the total number of people using the park [69].

We have identified limitations of this study to be addressed in future research. First, the focus on three joint-use parks of similar characteristics in central Texas limited the generalizability of findings. Moving forward, researchers can expand the number of joint-use parks to observe and select parks that differ by region, populations served, amenities, facilities, and other contextual elements. Second, we adapted SOPARC based on the traditional instrument in which data are collected on separate observational scans (e.g., physical activity of females and race/ethnicity of females) [70], preventing us from measuring children’s physical activity levels and adherence to COVID-19 protective behaviors simultaneously. We advise researchers to measure physical activity levels and adherence to COVID-19 behaviors together, following an adapted SOPARC protocol that exhibited relatively high interobserver reliability when assessing concurrent measures [71]. Third, we determined whether children wore face coverings at the group level unlike the individual-level observations for physical activity levels and social distancing. Subsequent studies can conduct separate observational scans for whether males and females wore face coverings. Fourth, we evaluated children’s adherence to COVID-19 protective behaviors without identifying whether individuals in proximity to each child resided in the same household. The Stay Home, Mask, and Otherwise Be Safe order in Austin had exceptions for social distancing and face covering guidelines when individuals were only in the presence of members of their own household [44]. Administering a brief survey to park users can assist researchers with identifying who resides in which household. Lastly, SOPARC methodology is limited to best estimations of what trained staff directly observe. There may be instances of misclassification such as incorrectly estimating whether an observed child is within the age group of interest (1–12).

## 5. Conclusions

Using direct observation at joint-use parks in economically disadvantaged Latinx communities in Austin, Texas, we established significant reductions in the number of overall children and number of children participating in physical activity during the COVID-19 pandemic compared to before the pandemic. These declines may be attributed to the COVID-19 order, changes in physical activity behavior of children, and impacts of the pandemic on guardians of children. We also observed the majority of children did not practice social distancing or wear face coverings when near others during the pandemic, potentially due to COVID-19 not perceived as a health concern and/or evolving and competing COVID-19 orders at both the city- and school district-level. Our findings suggest economically disadvantaged Latinx communities did not utilize joint-use parks as a health resource during the pandemic, and most of those who did visit parks may have increased viral transmission by not adhering to COVID-19 protective health behaviors. The city officials should consider how parks and other health infrastructure can be used safely and how to communicate this health and safety information to the public appropriately when COVID-19 and other communicable diseases threaten community health.

## Figures and Tables

**Table 1:** Pooled summary statistics for joint-use parks outside of school hours.

	Before COVID-19		During COVID-19	
Variables ^1^	Mean (Std. Dev.)	% of Total (Count)	Mean (Std. Dev.)	% of Total (Count)
Female children (#)	5.76 (4.77)	36.6% (311)	3.02 (3.17)	45.2% (163)
Male children (#)	9.96 (15.44)	63.4% (538)	3.67 (3.59)	54.8% (198)
Female children—sedentary (#)	1.81 (2.53)	31.5% (98)	0.83 (1.49)	27.6% (45)
Female children—light-to-moderate physical activity (#)	3.15 (2.85)	54.7% (170)	1.44 (1.81)	47.9% (78)
Female children—vigorous physical activity (#)	0.80 (1.63)	13.8% (43)	0.74 (1.42)	24.5% (40)
Male children—sedentary (#)	2.44 (4.31)	24.5% (132)	0.93 (1.30)	25.3% (50)
Male children—light-to-moderate physical activity (#)	5.54 (9.97)	55.6% (299)	1.59 (1.79)	43.4% (86)
Male children—vigorous physical activity (#)	1.98 (4.28)	19.9% (107)	1.15 (2.10)	31.3% (62)
Weekend (1 = weekend day)	0.17 (0.38)	16.7%(3)	0.17 (0.38)	16.7% (3)
Rain during observation (1 = rain)	0.02 (0.14)	1.90% (1)	0.06 (0.23)	5.60% (3)
Average heat index 16:00–18:30 (°C)	30.46 (8.15)	–	26.72 (5.34)	–

^1^ Day-level.

**Table 2:** Model output for relations between COVID-19 pandemic and use of joint-use parks by children outside of school hours.

Variables	(1) Female Users	(2) Male Users	(3) Female PA	(4) Male PA
Pandemic (1 = during pandemic) ^1^	0.54 ** (0.37–0.80)	0.38 *** (0.24–0.61)	0.58 ** (0.41–0.84)	0.40 *** (0.25–0.64)
Weekend (1 = weekend day) ^1^	1.15 (0.70–1.90)	0.76 (0.40–1.46)	1.00 (0.63–1.61)	0.82 (0.42–1.57)
Rain during observation (1 = rain) ^1^	0.06 * (0.01–0.54)	0.20 * (0.05–0.81)	0.00 (0.00–0.00) ^2^	0.16 * (0.03–0.79)
Average heat index 16:00–18:30 (°C) ^1^	1.00 (0.99–1.02)	0.99 (0.97–1.01)	1.00 (0.98–1.01)	0.99 (0.97–1.01)
Intervention park (1 = intervention)	0.79 (0.50–1.26)	0.86 (0.47–1.58)	0.83 (0.53–1.29)	0.82 (0.45–1.51)
Low-green park (1 = low-green)	1.21 (0.77–1.89)	1.38 (0.76–2.50)	1.70 * (1.12–2.57)	1.90 * (1.06–3.40)
Lnalpha	0.68 (0.41–1.02)	1.20 (0.87–1.66)	0.47 ** (0.28–0.79)	1.13 (0.80–1.59)
Constant	5.35 * (1.35–21.23)	17.98 *** (3.72–86.97)	3.68 * (1.01–13.36)	14.23 *** (2.96–68.44)
*N*	108	108	108	108

Coefficients; 95% confidence intervals in parentheses; * *p* < 0.05, ** *p* < 0.01, *** *p* < 0.001. Dependent variables: (1) daily number of female children; (2) daily number of male children; (3) daily number of physically active female children; (4) daily number of physically active male children. ^1^ Day-level. ^2^ Small non-zero estimates attributed to all days with rain having no observations of female children participating in physical activity.

**Table 3:** Adherence to COVID-19 protective health behaviors of children at joint-use parks outside of school hours.

Variables	% of Total (Count)
Female children who maintained social distance (≥ six feet)	39.4% (61)
Male children who maintained social distance (≥ six feet)	26.4% (47)
Observations where all children wore face coverings	6.0% (14)
Observations where some children wore face coverings	2.2% (5)
Observations where no children wore face coverings	91.8% (213)

## Data Availability

Data used and analyzed in the current study are available from the corresponding author upon reasonable request.
